# Biological Validation of Novel Polysubstituted Pyrazole Candidates with *in Vitro* Anticancer Activities

**DOI:** 10.3390/molecules21030271

**Published:** 2016-02-26

**Authors:** Hoda H. Fahmy, Nagy M. Khalifa, Magda M. F. Ismail, Hend M. El-Sahrawy, Eman S. Nossier

**Affiliations:** 1 Department of Therapeutical Chemistry, Pharmaceutical and Drug Industries Division, National Research Centre, Giza 12622, Egypt; hh_fahmy@yahoo.com; 2 Pharmaceutical Chemistry Department, Drug Exploration & Development Chair (DEDC), College of Pharmacy, King Saud University, Riyadh 11451, Saudi Arabia; 3 Department of Pharmaceutical Chemistry, Faculty of Pharmacy (Girls), Al-Azhar University, Cairo 11754, Egypt; m.elalfy101@gmail.com (M.M.F.I.); hendelsehrawi@hotmail.com (H.M.E.); emy28_s@hotmail.com (E.S.N.)

**Keywords:** 1,3,4-polysubstituted pyrazole derivatives, anticancer agents, synthesis

## Abstract

With the aim of developing novel antitumor scaffolds, a novel series of polysubstituted pyrazole derivatives linked to different nitrogenous heterocyclic ring systems at the C-4 position were synthesized through different chemical reactions and characterized by means of spectral and elemental analyses and their antiproliferative activity against 60 different human tumor cell lines was validated by the U.S. National Cancer Institute using a two stage process. The *in vitro* anticancer evaluation revealed that compound **9** showed increased potency toward most human tumor cell lines with GI_50_MG-MID = 3.59 µM, as compared to the standard drug sorafenib (GI_50_ MG-MID = 1.90 µM). At the same time, compounds **6a** and **7** were selective against the HOP-92 cell line of non-small cell lung cancer with GI_50_ 1.65 and 1.61 µM, respectively.

## 1. Introduction

The development of new antitumor agents is an important field of scientific activity, due to the toxic side effect problems of recent drugs. Many of the obtainable anticancer agents display unwanted side effects such as reduced bioavailability, toxicity and drug-resistance [1,2,3,4,5]. Incorporation of a pyrazole ring into different heteroaryl ring systems results in significant anticancer activities [6,7,8,9]. Different substituted pyrazole compounds have also been examined for their antiproliferative activities *in vitro* and antitumor activity *in vivo*, resulting in promising target products [10,11,12]. On the other hand, compounds containing pyrazole derivatives represent an advantageous choice for the synthesis of compounds with a broad spectrum of pharmacological activities, including anti-inflammatory [13], antibacterial, antifungal [14], inhibition of cyclooxygenase-2 [15], antiangiogenic [16], antipyretic [17], antihypertensive [18], antiplatelet [19], nitric oxide synthase (NOS) inhibitors [20] and anticancer activities [21]. Based on these observations and in continuation of our research on biologically active heterocycles [22,23,24,25], it was of interest to incorporate the 1,2,4-polysubstituted pyrazole ring system into different heteroaryl ring systems in one molecule in an attempt to obtain a new target anticancer agents.

## 2. Results and Discussion

### 2.1. Chemistry

**Scheme 1:**
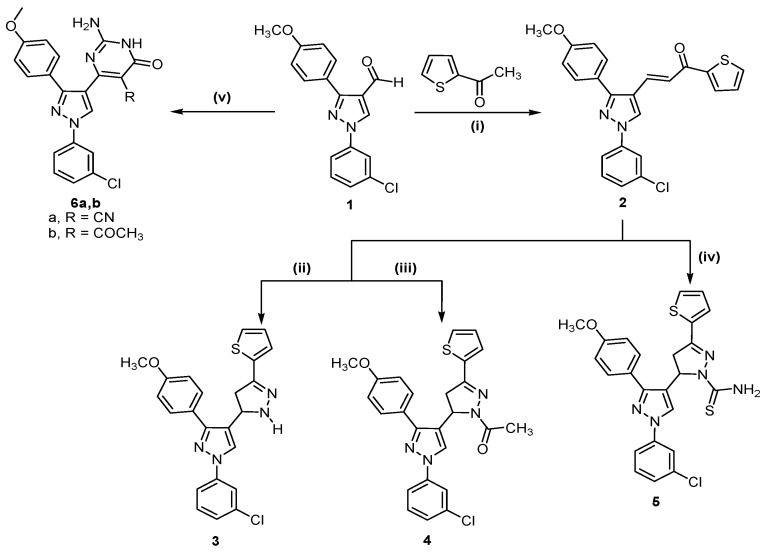
Synthetic route for trisubstituted pyrazole compounds **2**–**6**.

**Scheme 2:**
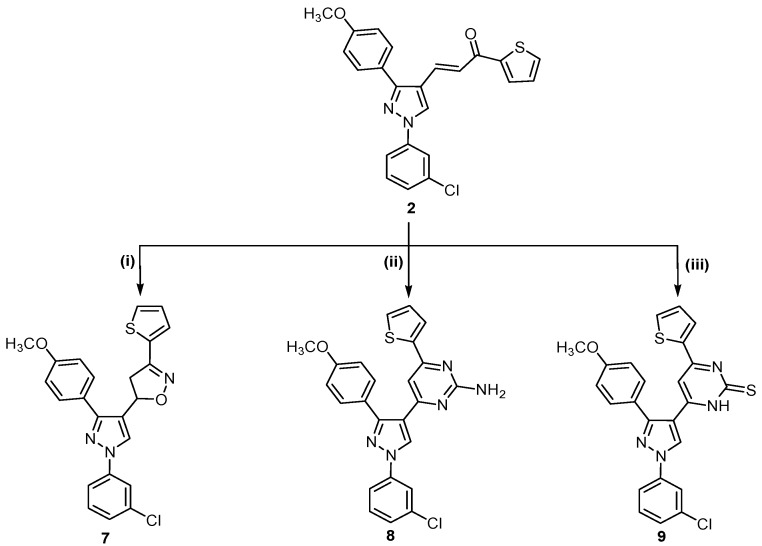
Synthetic route for trisubstituted pyrazole compounds **7**–**9**.

The reaction sequences outlined in Scheme 1 and Scheme 2 were used for the synthesis of the target compounds. Application of the Claisen Schmidt condensation on 2-acetylthiophene and 1-(3-chlorophenyl)-3-(4-methoxyphenyl)-1*H*-pyrazole-4-carboxaldehyde (**1**) in ethanolic sodium hydroxide solution according to literature methods [26,27] afforded (*E*)-3-(1-(3-chlorophenyl)-3-(4-methoxyphenyl)-1*H*-pyrazol-4-yl)-1-(thiophen-2-yl)prop-2-en-1-one (**2**), which was used as starting material. Cyclocondensation of the α,β-unsaturated ketone **2** with hydrazine hydrate in absolute ethanol or glacial acetic acid yielded the corresponding pyrazoline derivative **3** and N-acetyl-pyrazoline derivative **4**, respectively. On the other hand, heating of **2** with thiosemicarbazide in ethanolic NaOH gave 1-thiocarbamoyl pyrazole derivative **5**. In addition, condensation of compound **1** with ethyl cyanoacetate, or ethyl acetoacetate in the presence of guanidine hydrochloride gave 2-amino-5-cyano/acetyl-6-hydroxy-4-aryl pyrimidines **6a**,**b**, respectively (Scheme 1).

Finally, α,β-unsaturated ketone **2** was reacted with hydroxylamine hydrochloride in refluxing ethanol in the presence of sodium hydroxide as alkaline medium to afford the corresponding isoxazoline **7**. Treatment of **2** with guanidine sulfate in ethanolic sodium hydroxide gave 2-aminopyrimidine derivative **8**, which was reacted with thiourea in the presence of sodium hydroxide to give the corresponding pyrimidine-2-thione derivative **9** (Scheme 2).

### 2.2. In Vitro Anticancer Screening

The target compounds were selected by the U.S. National Cancer Institute (NCI), for anticancer activity screening. The screening is a two-stage process, beginning with the evaluation at a single dose (10 µM) and the compounds which display significant growth inhibition are then evaluated at five concentration levels. The first screening, where the selected compounds are evaluated at a single dose (10 µM) and the culture is incubated for 48 h, utilizes 60 different human tumor cell lines, representing leukemia, melanoma and cancers of lung, colon, central nervous system (CNS), ovary, kidney, prostate as well as breast.

The percentages of growth of the tested compounds against the full 60-cell line panel are listed in Table 1. The one dose mean graphs of the selected compounds revealed that compounds **6a**, **7** and **9** showed increased potency against most human cancer cell lines, so these compounds were selected for further evaluation at five dose concentration levels (0.01–100 µM).Regarding sensitivity against individual cell lines, compound **9** showed potent anticancer activity against all human cancer cell lines with GI_50_ 1.9–5.50 µM. It had the highest selectivity against the non-small cell lung cancer cell line EKVX, with GI_50_ 1.9 µM. At the same time, compounds **6a** and **7** showed the highest activity against the cell line HOP-92 belonging to the non-small cell lung cancer class with GI_50_ 1.7 and 1.6 µM, respectively, and against the renal cancer cell line A498 with GI_50_ 1.47 and 1.81 µM, respectively.

Examination of all the ligand structural modifications performed at the 4-position of 1,3,4-trisubstituted pyrazole scaffold to establish the structure activity relationships with anticancer acitivity shows the following results: first, introduction of the pyrimidine-2(1*H*)-thione moiety in the 4-position of the pyrazole moiety in compound **9** enhanced the potency towards most cancer cell lines. It has GI_50_ MG-MID = 3.6 µM against all subpanel tumor cell lines, comparable to that of sorafenib (GI_50_ MG-MID = 1.90 µM. Concerning the pyrazolyl derivative **6a**, it was observed that the activity was enhanced in 2-amino-6-oxopyrimidine-5-carbonitrile, which suggests that the polar 6-aminopyrimidine moiety has a role in enhancing anticancer activity (GI_50_ MG-MID = 5.70 µM), but less so than the 2-mercaptopyrimidine group. Finally, introduction of a 1-carbothioamide to a pyrazole or isoxazoline group substituted on the pyrazole greatly reduces the activity and these compounds showed the least potent activity. The results are presented in Table 1, Table 2, Table 3, Table 4, Table 5 and Table 6.

## 3. Experimental Section

### 3.1. General Information

Melting points were measured in open capillary tubes using a Griffin apparatus and are uncorrected. Structures of compounds were confirmed by routine spectrometric analysis. Elemental analyses were carried results were within ±0.4% of the theoretical values. Infrared spectra were recorded on a 435 IR spectrophotometer (Shimadzu Bruker, Tokyo, Japan) using KBr discs. ^1^H-NMR and ^13^C-NMR spectra were obtained on a Gemini 500 MHz spectrophotometer (Varian, Polo Alto, Ca, USA) or on a Bruker 500 MHz spectrophotometer, and measured in δ scale using TMS as an internal standard. Mass Spectra were recorded on a 5988 spectrometer (Hewlett Packard, California, USA). Analytical thin layer chromatography (TLC) was performed using silica gel aluminum sheets, 60 F_254_ (E. Merck, Darmstadt, Germany) for the progress of reactions and visualization with ultraviolet light (UV) at 365 and 254 nm.

### 3.2. 3-(1-(3-Chlorophenyl)-3-(4-methoxyphenyl)-1H-pyrazol-4-yl)-1-(thiophen-2-yl)prop-2-en-1-one (***2***)

A mixture of carbaldehyde derivative **1** (0.01 mol) and 2-acetylthiophene (0.01 mol) in of 30% ethanolic solution of NaOH (40 mL) was stirred for 12 h at room temperature. The progress of reaction was monitored by TLC. After completion, the reaction mixture was poured into acidified ice cold water of pH~2. The precipitated solid was filtered, washed with water and recrystallized to afford compound **2** in 87% yield; m.p. 144–146 °C (EtOH); IR (KBr) ν: 3079 (CH-Ar), 1641 (C=O), 1600 (C=C) cm^−1^; ^1^H-NMR (DMSO-*d*_6_): δ 3.81 (s, ^3^H, OCH_3_); 7.00–7.98 (m, ^12^H, ArH + CH=CH), 8.73 (s,^1^H, CH of pyrazole) ppm; ^13^C-NMR (DMSO-*d*_6_): δ 55.60, 114.34, 116.62, 117.75, 125.11, 125.95, 127.67, 129.08, 129.81, 131.74, 133.85, 134.47, 135.44, 141.08, 144.32, 151.54, 159.58, 191.96 ppm; MS (EI, 70 eV): *m*/*z* (%): 420 (11) [M]^+^; Anal. Calcd for C_23_H_17_ClN_2_O_2_S (420.91): C, 65.63; H, 4.07; N, 6.66; Found: C, 65.59; H, 4.16; N, 6.72.

### 3.3. 1-(3-Chlorophenyl)-4-(4,5-dihydro-3-(thiophen-2-yl)-1H-pyrazol-5-yl)-3-(4-methoxyphenyl)-1H-pyrazole *(**3**)*

To a solution of compound **2** (0.01 mol) in ethanol (30 mL) containing a catalytic amount of glacial acetic acid, a solution of hydrazine hydrate (98%, 0.5 mL) was added and the mixture was refluxed for 6 h. The reaction mixture was cooled to room temperature and the precipitated solid was filtered, dried and recrystallization provided compound **3** in 57% yield; m.p. 175–178 °C (EtOH); IR (KBr) ν: 3177 (NH), 1590 (C=C) cm^−1^; ^1^H-NMR (DMSO-*d*_6_): δ 2.99 (dd, ^1^H, CH), 3.70 (s, ^3^H, OCH_3_), 3.84 (dd, ^1^H, CH), 5.42 (dd, ^1^H, CH), 6.39–7.77 (m, ^11^H, Ar-H), 8.91 (s, ^1^H, CH of pyrazole), 11.52 (s, ^1^H, NH D_2_O exchangeable) ppm; ^13^C-NMR (DMSO-*d*_6_): δ 42.10, 55.61, 60.74, 114.22, 116.78, 117.76, 125.18, 125.90, 126.17, 127.38, 128.67, 129.65, 130.87, 131.26, 134.53, 137.55, 140.98, 151.24, 159.73, 158.76, 161.48 ppm; MS (EI, 70 eV): *m*/*z* (%): 434 (18) [M]^+^; Anal. Calcd for C_23_H_19_ClN_4_OS (434.94): C, 63.51; H, 4.40; N, 12.88; Found: C, 63.59; H, 4.53; N, 12.92.

### 3.4. 1-(5-(1-(3-Chlorophenyl)-3-(4-methoxyphenyl)-1H-pyrazol-4-yl)-4,5-dihydro-3-(thiophen-2yl)pyrazol-1-yl)-ethanone *(**4**)*

To a solution of compound **2** (0.01 mol) in glacial acetic acid (20 mL), hydrazine hydrate (0.01 mol) was added and the mixture was refluxed for 3 h. The reaction mixture was cooled to room temperature and the solid formed was filtered off, dried and recrystallized to get compound 4 in 65% yield; m.p. 150–154 °C (EtOH); IR (KBr) ν: 3080 (CH-Ar), 1677 (C=O), 1619 (C=N), 1588 (C=C) cm^−1^; ^1^H-NMR (DMSO-*d*_6_): 2.43 (s, ^3^H, COCH_3_), 3.30 (dd, ^1^H, CH), 3.81 (s, ^3^H, OCH_3_), 3.88 (dd, ^1^H, CH), 5.57 (dd, ^1^H, CH), 6.70–7.65 (m, ^11^H, Ar-H), 9.18 (s, ^1^H, CH of pyrazole) ppm; ^13^C-NMR (DMSO-*d*_6_): δ 24.01, 43.81, 55.60, 63.87, 114.26, 116.84, 117.62, 125.35, 125.86, 126.10, 127.56, 129.14, 129.78, 130.69, 131.08, 134.41, 137.82, 140.84, 150.39, 155.19, 159.12, 160.92, 168.19 ppm; MS (EI, 70 eV): *m*/*z* (%): 476 (43) [M]^+^; Anal. Calcd for C_25_H_21_ClN_4_O_2_S (476.98): C, 62.95; H, 4.44; N, 11.75; Found: C, 63.02; H, 4.35; N, 11.81.

### 3.5. 5-(1-(3-Chlorophenyl)-3-(4-methoxyphenyl)-1H-pyrazol-4-yl)-4,5-dihydro-3-(thiophen-2-yl)-pyrazole-1-carbothioamide *(**5**)*

To a mixture of chalcone **2** (0.01 mol) in absolute ethanol (30 mL), sodium hydroxide (1 g, 0.025 mol) was added. The reaction mixture was heated under reflux for 5 h. The contents were reduced, cooled and poured onto crushed ice. The resulting precipitate was collected by filtration and recrystallized to give in 61% yield; m.p. >300 °C (MeOH); IR (KBr) ν: 3407 (NH_2_), 1658 (C=N), 1523 (C=C), 1078 (C=S) cm^−1^; ^1^H-NMR (DMSO-*d*_6_): 3.13 (dd, ^1^H, CH), 3.84 (s, ^3^H, OCH_3_), 4.14 (dd, ^1^H, CH), 5.51 (dd, ^1^H, CH), 7.14–8.02 (m, ^11^H, Ar-H), 9.12 (s, ^1^H, CH of pyrazole), 9.93 (s, ^2^H, NH_2_ D_2_O exchangeable) ppm; ^13^C-NMR (DMSO-*d*_6_): δ 43.14, 55.64, 62.93, 114.33, 116.72, 117.58, 125.37, 125.80, 126.08, 127.54, 128.79, 129.22, 130.65, 131.16, 138.12, 137.82, 140.85, 151.23, 156.19, 159.30, 161.02, 176.55 ppm; MS (EI, 70 eV): *m*/*z* (%): 494 (6) [M]^+^; Anal. Calcd for C_24_H_20_ClN_5_OS_2_ (494.03): C, 58.35; H, 4.08; N, 14.18; Found: C, 58.27; H, 4.12; N, 14.23.

### 3.6. 2-Amino-4-(1-(3-chlorophenyl)-3-(4-methoxyphenyl)-1H-pyrazol-4-yl)-1,6-dihydro-6-oxopyrimidine-5-carbonitrile *(**6a**)* and 5-acetyl-2-amino-6-(1-(3-chlorophenyl)-3-(4-methoxyphenyl)-1H-pyrazol-4-yl)-pyrimidin-4(3H)-one *(**6b**)*

A mixture of compound **1** (0.01 mol), ethyl cyanoacetate or ethyl acetoacetate (0.01 mol) and (5 mL) of 40% ethanolic sodium hydroxide was stirred for 10 min, followed by addition of guanidine hydrochloride (0.01 mol) and the heating continued under refluxed for 3 h. The reaction mixture was diluted with ice-water and the formed precipitate was collected by filtration, washed several times with water, dried and recrystallized to afford the title compounds **6a**,**b**.

*2-Amino-4-(1-(3-chlorophenyl)-3-(4-methoxyphenyl)-1H-pyrazol-4-yl)-1,6-dihydro-6-oxopyrimidine-5-carbonitrile* (**6a**). Yield 64%; m.p. 144–148 °C (EtOH); IR (KBr) ν: 3341, 3145 (NH_2_, NH), 2219 (C≡N), 1663 (C=O), 1599 (C=C) cm^−1^; ^1^H-NMR (DMSO-*d*_6_): 2.85 (s, ^2^H, NH_2_ D_2_O exchangeable), 3.88 (s, ^3^H, OCH_3_), 7.03–8.75 (m, ^9^H, Ar-H and NH D_2_O exchangeable), 9.20 (s, ^1^H, CH of pyrazole) ppm; ^13^C-NMR (DMSO-*d*_6_): δ 55.63, 113.75, 115.31, 116.79, 117.77, 123.48, 125.21, 126.4, 127.94, 128.30, 129.82, 130.80, 134.57, 140.89, 155.04, 159.88, 160.60, 164.33, 171.88 ppm; MS (EI, 70 eV): *m*/*z* (%): 418 (14) [M]^+^; Anal. Calcd for C_21_H_15_ClN_6_O_2_ (418.84): C, 60.22; H, 3.61; N, 20.07; Found: C, 60.26; H, 3.58; N, 20.15.

*5-Acetyl-2-amino-6-(1-(3-chlorophenyl)-3-(4-methoxyphenyl)-1H-pyrazol-4-yl)pyrimidin-4-(3H)-one* (**6b**). Yield 68%, m.p. 135–137 °C (EtOH); IR (KBr) ν: 3410, 3152 (NH_2_,NH), 1675 (C=O), 1589 (C=C) cm^−1^; ^1^H-NMR (DMSO-*d*_6_): 2.30 (s, ^2^H, NH_2_ D_2_O exchangeable), 2.39 (s, ^3^H, CH_3_), 3.86 (s, ^3^H, OCH_3_), 6.90–7.96 (m, ^9^H, Ar-H and NH exchangeable with D_2_O), 9.14 (s, ^1^H, CH of pyrazole) ppm; ^13^C-NMR (DMSO-*d*_6_): δ 24.38, 55.60, 113.72, 115.94, 116.85, 117.73, 125.10, 126.01, 128.36, 130.76, 132.95, 134.47, 141.05, 151.14, 154.78, 159.60, 160.82, 164.21, 165.38, 182.70 ppm; MS (EI, 70 eV): *m*/*z* (%): 435 (7) [M]^+^; Anal. Calcd for C_22_H_18_ClN_5_O_3_ (435.86): C, 60.62; H, 4.16; N, 16.07; Found: C, 60.59; H, 4.11; N, 16.12.

### 3.7. 1-(3-Chlorophenyl)-3-(4-methoxyphenyl)-4-(3-(thiophen-2-yl)isoxazol-5-yl)-1H-pyrazole *(**7**)*

A mixture of compounds **2** (0.01 mol) and hydroxylamine hydrochloride (0.01 mol) in ethanol (30 mL) containing sodium hydroxide solution (0.5 g NaOH in 0.5 mL water) was refluxed for 3 h. The reaction mixture was poured onto ice-water, neutralized with drops of conc. Hydrochloric acid and the solid precipitate formed filtered off, washed with water and recrystallized to yield the desired compound **7** in 73% yield; m.p. >300 °C (EtOH); IR (KBr) ν: 3064 (CH-Ar), 1601 (C=C) cm^−1^; ^1^H-NMR (DMSO-*d*_6_): 3.99 (s, ^3^H, OCH_3_), 7.30 (d, ^2^H, H *J* = 20), 7.60 (d, ^1^H, CH); 7.75–8.45 (m, ^10^H, Ar-H), 9.15 (s, ^1^H, CH of pyrazole) ppm; ^13^C-NMR (DMSO-*d*_6_): δ 43.19, 55.61, 73.10, 114.38, 116.72, 117.68, 125.68, 125.84, 126.10, 127.43, 129.07, 129.57, 130.81, 131.29, 134.47, 138.21, 140.85, 151.20, 154.63, 160.92, 162.89 ppm; MS (EI, 70 eV): *m*/*z* (%): 433 (12) [M]^+^; Anal. Calcd for C_23_H_16_ClN_3_O_2_S (433.91): C, 63.66; H, 3.72; N, 9.68; Found: C, 63.74; H, 3.79; N, 9.71.

### 3.8. 4-(1-(3-Chlorophenyl)-3-(4-methoxyphenyl)-1H-pyrazol-4-yl)-6-(thiophen-2-yl)pyrimidin-2-amine *(**8**)*

An aqueous solution 5 mL of 40% sodium hydroxide was added gradually during a period of 3 h to a mixture of chalcone **2** (0.01 mol) and guanidine sulfate (0.01 mol) in ethanol (25 mL). The reaction mixture was refluxed for 5 h and the reaction was monitored by TLC. After completion of the reaction, the reaction mixture was poured onto ice-cold water and the solid product formed was collected by filtration, washed with water then recrystallized to get compound **8** in 67% yield; m.p. >300 °C (MeOH); IR (KBr) *ν*: 3347 (NH_2_); 1632 (C=N); 1589 (C=C) cm^−1^; ^1^H-NMR (DMSO-*d*_6_): 3.80 (s, ^3^H, OCH_3_), 6.68-8.08 (t, ^12^H, Ar-H), 8.93 (s,^1^H, CH of pyrazole), 10.18 (s, ^2^H, NH_2_ D_2_O exchangeable) ppm; ^13^C-NMR (DMSO-*d*_6_): δ 55.64, 82.10, 114.31, 116.80, 117.59, 125.30, 125.78, 126.09, 127.41, 128.67, 129.48, 130.79, 131.16, 134.49, 139.86, 141.05, 150.88, 152.10, 160.37, 164.21, 166.58, 164.25 ppm; Anal. Calcd for C_24_H_18_ClN_5_OS (459.95): C, 62.67; H, 3.94; N, 15.23; Found: C, 62.63; H, 3.88; N, 15.29.

### 3.9. 6-(1-(3-Chlorophenyl)-3-(4-methoxyphenyl)-1H-pyrazol-4-yl)-4-(thiophen-2-yl)pyrimidine-2-(1H)-thione *(**9**)*

A solution of the chalcone **2** (0.01 mol), thiourea (0.01 mol) and sodium hydroxide (0.1 g) in absolute ethanol (30 mL) was refluxed for 6 h. The reaction mixture was concentrated under vacuum, cooled and neutralized with dilute HCl. The formed product was filtered off, washed with water and recrystallized to get compound **9** in 76% yield; m.p. >300 °C (MeOH); IR (KBr) ν: 3422 (NH), 1595 (C=C), 1176 (C=S) cm^−1^; ^1^H-NMR (DMSO-*d*_6_): 3.82 (s, ^3^H, OCH_3_), 6.90–8.60 (m, ^12^H, Ar-H), 9.20 (s, ^1^H, CH of pyrazole), 9.95 (s, ^1^H, NH D_2_O exchangeable) ppm; ^13^C-NMR (DMSO-*d*_6_): δ 55.63, 104.56, 113.82, 115.89, 117.66, 125.38, 125.70, 126.03, 127.38, 128.34, 129.45, 130.77, 131.09, 134.42, 138.17, 140.95, 150.76, 157.21, 160.85, 161.80, 162.46, 184.33 ppm; MS (EI, 70 eV): 477 (8) [M]^+^; Anal. Calcd for C_24_H_17_ClN_4_OS_2_ (477): C, 60.43; H, 3.59; N, 11.75; Found: C, 60.38; H, 3.66; N, 11.82.

### 3.10. Measurement of Anticancer Activity

The experimental method used in anticancer screening has been adopted by U.S. National Cancer Institute according to reported standard procedure [28,29,30].

## 4. Conclusions

In summary, we have synthesized a series of novel pyrazole derivatives incorporated different heteroaryl ring systems in one molecule and evaluated these compounds for their anticancer activities against different 60 human cancer cell lines representing leukemia, melanoma and cancers of lung, colon, brain, ovary, breast, prostate and kidney cancer using a two-stage process. The pyrimidine-2(1*H*)-thione derivative **9** showed good anticancer activity with (GI_50_ MG-MID = 3.59 µM) compared to the standard drug sorafenib. The structures of the new compounds were elucidated using spectroscopic and elemental analysis.

## Figures and Tables

*Reagents and Conditions*: (i) ethanol/NaOH/rt./12 h, 87%; (ii) N_2_H_4_.H_2_O/ethanol/reflux/6 h, 57%; (iii) N_2_H_4∙_H_2_O/AcOH/reflux/3h, 65%; (iv) NH_2_NHCSNH_2_/ethanol/NaOH/reflux/5 h, 61%; (v) NC-CH2COOEt or CH_3_COCH_2_COOEt, H_2_N-C(NH)-NH_2_∙HCl/ethanol/NaOH/reflux 3 h, 64–68%.

*Reagents and Conditions*: (i) NH_2_OH∙HCl/ethanol/NaOH/reflux/3 h, 73%; (ii) H_2_N-C(NH)-NH_2_∙H_2_SO_4_/ethanol/NaOH reflux/5 h, 67%; (iii) NH_2_CSNH_2_/ethanol/NaOH/reflux/6 h, 76%.

**Table 1:** The mean growth percent of compounds **2**, **3**, **4**, **6a**, **6b**, **7**, **8** and **9**.

Cpd. No.	NSC No.	Mean Growth Percent
**2**	762925/1	95.56
**3**	763580/1	102.40
**4**	763581/1	98.43
**6a**	762930/1	67.27
**6b**	762931/1	76.45
**7**	762927/1	47.83
**8**	762928/1	99.26
**9**	762929/1	49.53

**Table 2:** GI_50_ (μM) of five-dose screening results of compounds **6a**, **7** and **9**.

Subpanel Cell Lines			
GI_50_			
Cell lines	6a	7	9
**Leukemia**			
**CCRF-CEM**	3.65	3.84	2.80
**HL-60(TB)**	3.96	4.73	3.49
**K-562**	3.45	3.43	2.91
**MOLT-4**	4.80	2.96	3.03
**RPMI-8226**	5.63	5.33	2.42
**SR**	3.73	3.03	4.00
**Non-Small Cell Lung Cancer**			
**A549/ATCC**	5.13	3.56	2.29
**EKVX**	3.29	2.07	1.93
**HOP-62**	10.1	13.90	7.14
**HOP-92**	1.65	1.61	2.20
**NCI-H226**	6.23	4.11	3.16
**NCI-H23**	5.44	3.82	3.43
**NCI-H460**	6.00	3.42	3.01
**NCI-H522**	3.11	4.27	2.54
**NCI-H322M**			
**Colon Cancer**			
**COLO 205**	7.65	3.21	2.35
**HCC-2998**	5.87	3.47	4.03
**HCT-116**	4.35	3.03	2.38
**HCT-15**	5.07	4.48	3.69
**HT29**	4.29	3.85	3.82
**KM12**	3.84	2.98	3.67
**SW-620**	4.38	4.35	3.79
**CNS Cancer**			
**SF-268**	5.86	4.94	4.52
**SF-295**	2.36	2.70	2.07
**SF-539**	6.59	7.47	4.70
**SNB-19**	4.58	6.08	4.93
**SNB-75**	2.88	3.52	3.49
**U251**	3.66	3.61	2.60
**Melanoma**			
**LOX IMVI**	4.02	3.04	3.56
**MALME-3M**	5.83	2.60	3.49
**M14**	3.25	3.30	2.81
**MDA-MB-435**	2.16	3.26	3.13
**SK-MEL-2**	3.94	3.34	2.87
**SK-MEL-28**	4.98	4.93	3.93
**SK-MEL-5**	4.07	4.74	2.93
**UACC-257**	5.69	3.07	3.06
**UACC-62**	3.33	3.55	2.78
**Ovarian Cancer**			
**IGROV1**	3.01	4	3.61
**OVCAR-3**	3.38	5.07	3.04
**OVCAR-4**	7.71	4.48	3.47
**OVCAR-5**	14.60	7.63	6.38
**OVCAR-8**	10.10	4.04	3.15
**NCI/ADR-RES**	3.33	4.01	4.77
**SK-OV-3**	6.56	8.89	5.91
**Renal Cancer**			
**786-0**	4.65	3.39	4.18
**A498**	1.47	1.81	3.01
**ACHN**	9.92	4.39	4.05
**CAKI-1**	2.52	2.05	3.03
**RXF 393**	4.51	3.49	3.76
**SN12C**	6.41	3.48	3.92
**TK-10**	13.50	5.21	4.55
**UO-31**	2.94	2.56	2.82
**Prostate Cancer**			
**PC-3**	6.30	3.2	3.09
**DU-145**	17.9	7.08	5.49
**Breast Cancer**			
**MCF7**	3.54	3.53	3.02
**MDA-MB-231/ATCC**	3.39	3.08	3.52
**HS 578T**	3.55	4.42	4.26
**BT-549**	3.55	4.99	3.18
**T-47D**	6.05	3.86	4.16
**MDA-MB-468**	2.12	2.68	2.57

GI_50_: (growth inhibitory activity) the drug concentration that reduces cellular growth by 50%.

**Table 3:** TGI (μM) of five-dose screening results of compounds **6a**, **7** and **9**.

Subpanel Cell Lines			
TGI			
Cell lines	6a	7	9
**Leukemia**			
**CCRF-CEM**	33.80	>100	>100
**HL-60(TB)**	14.20	38.30	13.20
**K-562**	>100	>100	>100
**MOLT-4**	39.80	19.50	25.20
**RPMI-8226**	55.50	61.90	10.90
**SR**	21.20	12.10	31.90
**Non-Small Cell Lung Cancer**			
**A549/ATCC**	50.80	24.50	16.50
**EKVX**	35.40	13.50	21.70
**HOP-62**	26.20	28.10	22.10
**HOP-92**	12.20	8.13	10.80
**NCI-H226**	70.300	30.30	54.40
**NCI-H23**	46.20	20.80	16.20
**NCI-H460**	27.70	16.60	11.00
**NCI-H522**	9.80	18.40	8.86
**NCI-H322M**			
**COLO 205**	20.90	7.24	5.59
**HCC-2998**	24.90	14.60	16.40
**HCT-116**	16.20	12.70	11.20
**HCT-15**	>100	32.90	>100
**HT29**	20.00	15.10	13.00
**KM12**	15.20	11.10	14.40
**SW-620**	34.20	22.60	22.30
**CNS Cancer**			
**SF-268**	35.4	47.10	73.60
**SF-295**	9.22	13.20	7.09
**SF-539**	32.2	36.60	19.70
**SNB-19**	100	>100	>100
**SNB-75**	21.1	34.70	25.60
**U251**	18.8	15.40	12.40
**Melanoma**			
**LOX IMVI**	20.10	10.90	13.80
**MALME-3M**	40.60	10.60	15.80
**M14**	34.00	17.10	14.00
**MDA-MB-435**	5.48	17.30	19.00
**SK-MEL-2**	15.80	10.90	6.90
**SK-MEL-28**	27.90	17.80	15.70
**SK-MEL-5**	15.90	17.30	10.90
**UACC-257**	47.30	15.10	18.00
**UACC-62**	15.40	20.00	19.20
**Ovarian Cancer**			
**IGROV1**	18.10	16.20	15.30
**OVCAR-3**	10.00	25.00	9.42
**OVCAR-4**	61.90	36.40	>100
**OVCAR-5**	51.40	74.10	>100
**OVCAR-8**	60.30	36.00	>100
**NCI/ADR-RES**	15.90	38.10	41.80
**SK-OV-3**	30.70	44.10	36.80
**Renal Cancer**			
**786-0**	33.50	>100	>100
**A498**	6.75	14.60	17.00
**ACHN**	>100	>100	>100
**CAKI-1**	15.30	6.02	8.68
**RXF 393**	22.40	20.10	19.50
**SN12C**	>100	18.70	>100
**TK-10**	53.90	43.80	32.80
**UO-31**	47.80	11.80	26.50
**Prostate Cancer**			
**PC-3**	>100	36.90	>100
**DU-145**	70.10	35.80	72.50
**Breast Cancer**			
**MCF7**	28.50	16.90	33.40
**MDA-MB-231/ATCC**	53.30	16.90	26.00
**HS 578T**	40.30	45.50	>100
**BT-549**	22.40	21.30	15.90
**T-47D**	32.20	25.30	34.60
**MDA-MB-468**	6.43	12.30	9.91

TGI: the drug concentration required for total growth inhibition.

**Table 4:** LC_50_ (μM) of five-dose screening results of compounds **6a**, **7** and **9**.

Subpanel Cell Lines			
LC_50_			
Cell lines	6a	7	9
**Leukemia**			
**CCRF-CEM**	>100	>100	>100
**HL-60(TB)**	>100	>100	>100
**K-562**	>100	>100	>100
**MOLT-4**	>100	>100	>100
**RPMI-8226**	>100	>100	>100
**SR**	>100	>100	>100
**Non-Small Cell Lung Cancer**			
**A549/ATCC**	>100	>100	>100
**EKVX**	>100	>100	>100
**HOP-62**	67.80	56.90	59.80
**HOP-92**	>100	>100	83.20
**NCI-H226**	>100	>100	>100
**NCI-H23**	>100	>100	>100
**NCI-H460**	>100	>100	52.00
**NCI-H522**	>100	93.50	>100
**NCI-H322M**			
**Colon Cancer**			
**COLO 205**	>50	24.70	40.00
**HCC-2998**	>100	64.70	96.00
**HCT-116**	46.00	40.60	33.80
**HCT-15**	>100	>100	>100
**HT29**	>100	>100	46.90
**KM12**	49.70	84.60	49.30
**SW-620**	>100	>100	>100
**CNS Cancer**			
**SF-268**	>100	>100	>100
**SF-295**	84.70	>100	61.30
**SF-539**	>100	>100	82.30
**SNB-19**	>100	>100	>100
**SNB-75**	>100	>100	>100
**U251**	82.80	49.50	38.30
**Melanoma**			
**LOX IMVI**	74.10	54.90	45.20
**MALME-3M**	>100	60.00	97.20
**M14**	>100	84.10	90.10
**MDA-MB-435**	>100	>100	>100
**SK-MEL-2**	99.10	66.80	35.60
**SK-MEL-28**	>100	52.40	58.80
**SK-MEL-5**	51.30	47.50	41.90
**UACC-257**	>100	>100	>100
**UACC-62**	53.30	65.70	71.60
**Ovarian Cancer**			
**IGROV1**	>100	71.20	73.80
**OVCAR-3**	60.30	>100	35.90
**OVCAR-4**	>100	>100	>100
**OVCAR-5**	>100	100	>100
**OVCAR-8**	>100	>100	>100
**NCI/ADR-RES**	>100	>100	>100
**SK-OV-3**	>100	>100	>100
**Renal Cancer**			
**786-0**	>100	>100	>100
**A498**	>100	45.20	73.20
**ACHN**	>100	>100	>100
**CAKI-1**	>100	75.90	>100
**RXF 393**	>100	>100	>100
**SN12C**	>100	>100	>100
**TK-10**	>100	>100	>100
**UO-31**	>100	>100	>100
**Prostate Cancer**			
**PC-3**	>100	>100	>100
**DU-145**	>100	>100	>100
**Breast Cancer**			
**MCF7**	>100	83.60	>100
**MDA-MB-231/ATCC**	>100	>100	>100
**HS 578T**	>100	>100	>100
**BT-549**	>100	66.30	>100
**T-47D**	>100	>100	>100
**MDA-MB-468**	>100	>100	>100

LC_50_: the drug concentration required for killing 50% of cells.

**Table 5:** Median growth inhibitory concentrations (GI_50_, μM) of *in vitro* subpanel tumor cell lines and GI_50_ (μM) full panel mean-graph mid-points (MG-MID) of compounds **6a**, **7** and **9** in comparison with sorafenib.

Subpanel Tumor Cell Lines										
Cpd. No.	Leukemia	Lung	Colon	CNS	Melanoma	Ovarian	Renal	Prostate	Breast	GI_50_ MG-MID
**6a**	4.2	5.1	5.1	4.3	4.1	7.0	5.7	12.1	3.7	5.7
**7**	3.9	4.6	3.6	4.7	3.5	5.5	3.3	5.1	3.8	4.2
**9**	3.1	3.2	3.4	3.7	3.2	4.3	3.7	4.3	3.5	3.6
**Sorafenib**										1.9

**Table 6:** Selectivity ratios for compounds **6a**, **7** and **9** towards the nine tumor cell lines.

Subpanel Tumor Cell Lines									
Cpd. No.	Leukemia	Lung	Colon	CNS	Melanoma	Ovarian	Renal	Prostate	Breast
**6a**	1.4	1.1	1.1	1.3	1.4	0.8	1.0	0.5	1.5
**7**	1.1	0.9	1.2	0.9	1.2	0.8	1.3	0.8	1.1
**9**	1.2	1.1	1.1	1.0	1.1	0.8	1.0	0.8	1.0
